# The Testing Effect and Far Transfer: The Role of Exposure to Key Information

**DOI:** 10.3389/fpsyg.2016.01977

**Published:** 2016-12-26

**Authors:** Gerdien G. van Eersel, Peter P. J. L. Verkoeijen, Migle Povilenaite, Remy Rikers

**Affiliations:** ^1^Department of Psychology, Education and Child Studies, Faculty of Social Sciences, Erasmus University RotterdamRotterdam, Netherlands; ^2^Learning and Innovation Center, Avans University of Applied SciencesBreda, Netherlands; ^3^Roosevelt Center for Excellence in Education, University College Roosevelt, Utrecht UniversityMiddelburg, Netherlands

**Keywords:** testing effect, far transfer, cued recall, key information, feedback, retrieval practice

## Abstract

Butler (2010: Experiment 3) showed that retrieval practice enhanced transfer to a new knowledge domain compared to rereading. The first experiment of the present study was a direct replication of Butler’s third experiment. Participants studied text passages and then either reread them three times or went through three cycles of cued recall questions (i.e., retrieval practice) with feedback. As in Butler’s (2010) experiment, an advantage of retrieval practice on the final far transfer test emerged after 1 week. Additionally, we observed an advantage of retrieval practice on the final test administered after 5 min. However, these advantages might have been due to participants in the retrieval practice condition receiving focused exposure to the key information (i.e., the feedback) that was needed to answer the final test questions. We therefore conducted a second experiment in which we included the retrieval practice condition and the reread condition from our first experiment, as well as a new reread-plus-statements condition. In the reread-plus-statements condition, participants received focused exposure to the key information after they had reread a text. As in Experiment 1, we found a large effect on far transfer when retrieval practice was compared to rereading. However, this effect was substantially reduced when retrieval practice was compared to the reread-plus-statements condition. Taken together, the results of the present experiments demonstrate that Butler’s (2010) testing effect in far transfer is robust. Moreover, focused exposure to key information appears to be a significant factor in this far transfer testing effect.

## Introduction

Retrieving information from memory after an initial learning phase enhances long-term retention more than restudying the material; an advantage referred to as the (retrieval practice) testing effect (for reviews, see Delaney et al., 2010; Roediger and Butler, 2011; Carpenter, 2012; Karpicke, 2012; Karpicke et al., 2014; Rowland, 2014). The testing effect has been demonstrated with a variety of practice tests, materials, and age groups (Dunlosky et al., 2013). In most of the testing effect research, the materials in the intermediate test and the final test are identical (e.g., Roediger and Karpicke, 2006a; Roediger et al., 2011; Wooldridge et al., 2014). However, it is important to determine whether the testing effect still emerges when the final test is different from the intermediate test and measures related but new knowledge, i.e., whether *transfer* of knowledge takes place (Salomon and Perkins, 1989; McDaniel et al., 2009, 2013).

A relatively small but increasing number of studies has shown that retrieval practice benefits the transfer of knowledge (for a recent review, see Carpenter, 2012). Transfer may be broadly defined as the ability to apply previously learned knowledge or skills in a novel context (Carpenter, 2012). To assess transfer with respect to the testing effect, Carpenter (2012) makes a distinction between three dimensions along which the differences between learning and transfer contexts can be compared: temporal context, test format, and knowledge domain. Following Barnett and Ceci (2002), a transfer task can be evaluated on each of these dimensions in terms of the level of transfer (i.e., *near* vs. *far* transfer).

Many studies have found that the beneficial effect of retrieval practice transfers across temporal contexts, as the effect of retrieval practice usually occurs after a retention interval of some days (e.g., Chan et al., 2006; Carpenter et al., 2009; Johnson and Mayer, 2009). However, in nearly all retrieval practice studies, the test formats and knowledge domains are comparable between the learning phase and the test phase [e.g., Carpenter and DeLosh, 2006; Carpenter et al., 2006; Agarwal et al., 2008 (Exp. 1); Coppens et al., 2011]. Only a small number of studies has shown transfer of a retrieval practice effect across different test formats (e.g., Karpicke and Zaromb, 2010; Carpenter, 2011; Halamish and Bjork, 2011; Sensenig et al., 2011; Rowland and DeLosh, 2015), and across both different test formats and temporal contexts (e.g., Kang et al., 2007; Rohrer et al., 2010; Hinze and Wiley, 2011; Lyle and Crawford, 2011; Blunt and Karpicke, 2014; McDermott et al., 2014; Rawson et al., 2015). Yet in all of the previous studies – and in fact in the vast majority of retrieval practice studies – the knowledge domain is the same in the learning phase and the final test. To the best of our knowledge, there is only one study (Butler, 2010) in which the retrieval practice effect emerged on a final transfer test tapping onto a different knowledge domain. Specifically, Butler (2010: Experiment 3) found a positive effect of retrieval practice on a final test that consisted of questions pertaining to topics from a different knowledge domain than the intermediate test questions. Note that although in Carpenter’s (2012) review several papers are mentioned in the section called “Transfer across knowledge domains,” only the paper by Butler (2010) actually meets the condition stated in this title.

In Butler’s (2010) crucial third experiment, participants first read six prose texts, and then reread three of the passages and practiced cued recall on the other three passages. In the retrieval practice condition participants answered conceptual cued recall questions, and afterward they received feedback in the form of the correct response. One week later, participants took the final transfer test. This test consisted of questions from different knowledge domains than the questions presented during the practice phase. On the final test, participants performed better after cued recall than after rereading. Because Butler’s (2010) third experiment has been the only one to demonstrate that retrieval practice fosters transfer to a different knowledge domain, it is important to investigate whether Butler’s results are robust. In addition, Butler’s sample size was small (20 participants), with imprecise parameter estimations as a result. Now given the importance of replication in (psychological) science (e.g., Cartwright, 1991; Ioannidis, 2005; Open Science Collaboration, 2012; Pashler and Wagenmakers, 2012; Klein et al., 2014), the purpose of our first experiment was to conduct an exact replication (see Schmidt, 2009) of Butler’s third experiment.

Furthermore, the observed beneficial effect of retrieval practice may have been partly due to another factor, namely the focused exposure to key information (i.e., the feedback) in the retrieval practice condition versus the reread condition. To illustrate this point, consider this example of a cued recall question from the retrieval practice condition: “Some bats use echolocation to navigate the environment and locate prey. How does echolocation help bats to determine the distance and size of objects?” The answer, which was taken from the text and presented as feedback, was the following: “Bats emit high-pitched sound waves and listen to the echoes. The distance of an object is determined by the time it takes for the echo to return. The size of the object is calculated by the intensity of the echo: a smaller object will reflect less of the sound wave, and thus produce a less intense echo.” The related transfer question was the following: “Submarines use SONAR to navigate underwater much like bats use echolocation to navigate at night. Using SONAR, how does a submarine determine that an object is moving toward it (i.e., rather than away from it)?” (“Answer: The submarine can tell the direction that an object is moving by calculating whether the time it takes for the sound waves to return changes over time. If the object is moving toward the submarine, the time it takes the sound wave to return will get steadily shorter. Also, the intensity of the sound wave will increase because the object will reflect more of the sound wave as it gets closer.”).

This example demonstrates that the retrieval practice questions and the final tests questions were conceptually related; the same principles that were learned during retrieval practice needed to be applied to the final test questions in the different knowledge domains. This means that participants in the retrieval practice condition may have had an advantage compared to the reread condition because they had already seen the relevant principles in the form of the key information that was provided as feedback during retrieval practice. Although participants in the reread condition had also seen these principles when they reread the whole text, they had only seen them as a part of the full text that contained additional information, not as answers to specific questions. Hence, in the retrieval practice condition, participants only needed to retrieve the key information from the feedback in the learning phase and apply it to the final test questions. By contrast, in the reread condition participants had to retrieve and select the part of the text relevant to the problem, and apply it to the final test items. In the reread condition, it might have been difficult to determine which part of the text was relevant for a final test question. As a result, final test performance in the reread condition might have suffered compared to the retrieval practice condition.

We therefore carried out another experiment that was identical to our first experiment, but with an extra reread-plus-statements condition, besides the reread and the retrieval practice conditions. In the reread-plus-statements condition, participants reread a text, followed by focused exposure to key information. This information consisted of statements that contained the same information as the feedback in the retrieval practice condition. In this way, we tested whether the focused exposure to key information could – partly – account for the testing effect found in Butler (2010).

## Experiment 1

The first experiment was a direct replication of Butler’s (2010) third experiment, but with an extra 5-min retention interval. Participants first read six texts and then repeatedly reread three of the texts, and repeatedly took cued recall tests with immediate feedback on the other three texts. After 5 min or after 1 week, participants completed a final transfer test.

### Method

#### Participants

This experiment was carried out in accordance with the recommendations of the Ethical Committee of the Department DPECS at the Erasmus University Rotterdam, with written informed consent from all participants. Fifty-six people participated in the study and were rewarded with course credits. Their mean age was 19.71 (*SD* = 3.43). Sixteen of them were males, forty were females. All of the participants were Psychology undergraduates, with 18 of them having the Dutch nationality, while the others had (25) other nationalities. The non-Dutch participants were students of the English-taught international bachelor in Psychology, where they had been accepted on the basis of their scores on internationally accepted English language tests. The Dutch participants had been taught English for 8 years during primary and secondary education, and can be considered as highly proficient in written English.

#### Materials and Design

We used Butler’s (2010) original materials: six prose passages in English about different topics of between 550 and 600 words in length. Each passage included four concepts. For each concept, Butler (2010) had created a question to assess transfer to a different knowledge domain (see the Introduction for an example). Each transfer question required the application of a concept from the initial learning session. The correct response was between one and three sentences long. The experiment had a 2 Study Method (reread vs. retrieval practice) ^∗^ 2 Retention Interval (5 min vs. 1 week) mixed design with repeated measures on the first factor. Note that in our study, we added an additional 5-min retention interval to Butler’s (2010) original design, in order to observe whether the testing effect would increase over time, which is sometimes regarded as a defining feature of the testing effect (e.g., Roediger and Karpicke, 2006b; Delaney et al., 2010; Kornell et al., 2011). Participants were randomly assigned to the levels of the between-subjects factor. Like in Butler (2010), we used four counterbalanced versions of the experiment by combining two orders of initial learning condition with two orders of the passages. Also as in Butler’s experiment, the dependent variable was the proportion of correct answers to the 24 final test transfer questions. Following Simmons et al. (2011), we have reported all conditions and all measures in this experiment.

#### Procedure

The procedure was identical to the procedure of Butler’s (2010) third experiment. Our study was conducted on a computer using E-Prime software, and Butler had provided us with the original E-Prime files that he had used for his original study (2010). In the first session, participants read the six English prose texts for 2 min each. Afterward, they repeatedly (i.e., three times) reread three of the passages for 2 min each, and repeatedly (i.e., three times) took identical four-item cued recall tests with immediate feedback (retrieval practice) on the three other passages. In the retrieval practice condition, participants answered four conceptual cued recall questions per text, and received feedback in the form of the correct response after each question. There was no time limit to answer the questions or review the feedback. Participants were encouraged to think a while, and to generate a response to every question (Butler, personal communication, October 6, 2014). Half of the participants took the final test after 5 min, the other half after 1 week. The final test was self-paced and consisted of 24 transfer questions about different knowledge domains.

### Results

Following Kline (2004, Chapter 3) and Cumming (2014), we use the term ‘statistical’ instead of ‘significant’ for all statistical analyses, because the latter is often erroneously understood as meaning ‘important.’

#### Scoring

Two research assistants and the first author independently scored 27% of the answers to the final test questions. Each answer was scored as either correct or incorrect based on the correct answers provided in Butler’s (2010) supplemental material. Cohen’s kappa was used as the interrater reliability measure and was 0.82 for the 5-min condition and 0.74 for the 1-week condition. These coefficients indicate a substantial level of agreement (Landis and Koch, 1977). The remaining responses were scored by the first author. Note that Cohen’s kappa is based on the absolute agreement between raters. Such an agreement is unnecessarily strict when the aim is – like in this experiment – to evaluate the mean difference between groups on a dependent variable, rather than to obtain a reliable estimate of the absolute level of performance within each group. In the former case, it is sufficient that raters are *consistent* regarding their final test total scores, without absolute agreement. We therefore also calculated the Pearson correlation coefficient between the total final test scores given by two raters, which was *r* = 0.97, *p* < 0.001, for the 5-min condition and *r* = 0.93, *p* = 0.002, for the 1-week condition.

#### Retrieval Practice Tests

The proportion of correct responses to the initial cued recall tests increased in a curvilinear fashion from Test 1 (*M* = 0.39, *SD* = 0.20) to Test 2 (*M* = 0.77, *SD* = 0.18) to Test 3 (*M* = 0.84, *SD* = 0.14). A repeated measures ANOVA yielded a statistical main effect of Test Session, *F*(1.82,98.10) = 231.20, *MSE* = 0.02, *p* < 0.001, $\eta_{p}^{2}$ = 0.81, for which there was a linear trend, *F*(1,54) = 329.52, *MSE* = 0.02, *p* < 0.001, $\eta_{p}^{2}$ = 0.86, as well as a quadratic trend *F*(1,54) = 75.00, *MSE* = 0.01, *p* < 0.001, $\eta_{p}^{2}$ = 0.58. The mean proportion of correct responses during the learning phase did not differ between the 5-min condition (*M* = 0.69, *SD* = 0.13) and the 1-week condition (*M* = 0.64, *SD* = 0.15), *t*(53) = 1.16, *p* = 0.25, $\eta_{p}^{2}$ = 0.03, 95% CI of the difference [-0.03, 0.12]. Note that due to an error, the retrieval practice data of one participant were not included in these calculations.

#### Time on Task

During retrieval practice, there were three texts with four questions each. All questions were repeated three times, resulting in a total number of 36 questions. The distribution of the number of seconds that participants spent on answering a question during retrieval practice was skewed to the right, so we report both the mean and the median. The mean was 76.88 s (*SD* = 38.55) and the median 70.73 s. The number of seconds that participants spent on reading the feedback was also skewed to the right: the mean was 14.42 s (*SD* = 8.96) and the median 12.14 s. Taken together, the mean number of seconds participants spent on each question (responding and reading feedback) was 91.29 (median = 81.76). Because there were four questions per passage, it took participants on average 365.16 s to complete a test on each passage (median = 327.04 s). In the reread condition, all participants had 120 s to reread a passage. Note that due to an error, the time-on-task data of three participants were not included.

#### Final Tests

A 2 Study Method (reread vs. retrieval practice) ^∗^ 2 Retention Interval (5 min vs. 1 week) Repeated Measures ANOVA on the proportion of correct answers did not yield a statistical interaction effect between study method and retention interval, *F*(1,54) = 2.41, *MSE* = 0.02, *p* = 0.126, $\eta_{p}^{2}$ = 0.04 (**Table 1**). In accordance, the difference between retrieval practice and rereading was large after 5 min, *t*(27) = 6.98, *p* < 0.001, *Cohen’s d* = 1.32, 95% CI of the difference [0.18, 0.33], and was large after 1 week, *t*(27) = 5.11, *p* < 0.001, *Cohen’s d* = 0.97, 95% CI of the difference [0.11, 0.25]. In sum, after both retention intervals there was large advantage of retrieval practice over rereading. In addition, there was a statistical main effect of study method, *F*(1,54) = 73.66, *MSE* = 0.02, *p* < 0.001, $\eta_{p}^{2}$ = 0.58, 95% CI of the difference [0.17, 0.27], but not of retention interval, *F*(1,54) = 1.50, *MSE* = 0.06, *p* = 0.226, $\eta_{p}^{2}$ = 0.03, 95% CI of the difference [-0.04, 0.15].

**Table 1:** Proportion of Answers Correct in Experiment 1 by Retention Interval and Study Method.

	Retention interval	
	
Study method	Five minutes	One week
Retrieval practice	0.57 (0.04)	0.59 (0.04)
Reread	0.31 (0.03)	0.41 (0.03)


*Standard errors are between brackets.*

We also performed a Bayesian Repeated Measures ANOVA (Rouder et al., 2012, 2016) in the software program JASP (Love et al., 2015; Wagenmakers et al., 2016) with a default uniform distribution of prior model probabilities (for arguments for the Bayesian approach, e.g., Dienes, 2011). The Bayes factor for the interaction effect between study method and retention interval was BF01 = 1.38, showing that the likelihood of the data under the null hypothesis was 1.38 times the likelihood of the data under the alternative hypothesis. Following Wetzels and Wagenmakers (2012), this is anecdotal evidence for the null hypothesis that postulates the absence of the interaction effect. The Bayes Factor BF10 for the factor study method was larger than 100, indicating that the observed data were more than 100 times more likely under the alternative hypothesis than under the null hypothesis. Bayes Factors larger than 100 are decisive evidence in favor of the relevant hypothesis. In this experiment, this meant decisive evidence for the advantage of retrieval practice over rereading on the final transfer test. The Bayes Factor for the factor retention interval was BF01 = 2.02, which can be interpreted as anecdotal evidence in favor of the null hypothesis of no difference between the two intervals on the proportion of correct answers on the final transfer test.

The time-on-task differed considerably between the two conditions in our experiment, which might have confounded the final test results. To assess whether this was the case, we calculated the time-on-task difference between the retrieval practice condition and the reread condition for each participant. Because time-on-task in the reread condition was a constant, the variance of these time-on-task difference scores amounted to the variance of the time-on-task scores in the retrieval practice condition. Furthermore, for each participant we calculated a final test difference score by subtracting the transfer score in the reread condition from that in the retrieval practice condition. Subsequently, we correlated these two difference scores and found no trace of a statistical correlation between the time-on-task difference scores and the difference scores on the transfer test, *r* = 0.04, *p* = 0.765. This low and non-statistical correlation indicates that an increased time-on-task in the retrieval practice condition was not associated with a larger advantage of retrieval practice over reread, suggesting that the retrieval practice effect was not confounded by time-on-task differences between conditions.

### Discussion

Experiment 1 showed a large benefit of retrieval practice compared to rereading on the final far transfer test administered after 1 week, that is, a far transfer testing effect. Furthermore, the effect size associated with this testing effect was large (*Cohen’s d* = 0.97), which is comparable to Butler’s (2010) *Cohen’s d* of 1.17^1^. Hence, the results of Experiment 1 convincingly replicated the results of Butler’s (2010) third experiment. As such, our findings provide a crucial independent reinforcement of this important finding within the testing effect literature.

Note that the overall final test performance in Experiment 1 did not differ between the 5-min condition and the 1-week condition. The length and the nature of the experimental procedure might explain this remarkable result. It took on average almost 1.5 h (85 min) to complete the first session. At the end of the experiment, the experiment leader always asked how everything went, and whether the participant had any comments or questions. More than 50% of the participants reported that the experiment was tiring or boring. Hence, participants in the 5-min condition were probably not as motivated and concentrated to start the final test session as participants in the 1-week condition. This, in turn, might have resulted in comparable performance for both retention intervals. Moreover, although there was no difference in the number of skipped final test questions between the 1-week condition and the 5-min condition, participants in the 1-week condition (*M* = 32.84, *SD* = 11.56) took almost 7 min longer to complete the final test than participants in the 5-min condition (*M* = 25.94, *SD* = 8.43), *t*(50) = -2.46, *p* = 0.018, *r* = 0.33, again indicating that the latter might have been less motivated than the former.

The novel result of Experiment 1 was that the benefit of retrieval practice already emerged after a retention interval of 5 min. Although there is agreement in the literature that the testing effect usually arises after a long retention interval (i.e., longer than 1 day), mixed support has been found for short intervals (Rowland, 2014). A factor that can partly explain these mixed findings is feedback (Rowland, 2014). In several studies where feedback was provided after retrieval practice, testing effects did emerge after a short retention interval (e.g., Carrier and Pashler, 1992; Bishara and Jacoby, 2008; Carpenter et al., 2008; Kornell et al., 2009; Jacoby et al., 2010; Kang, 2010; Wartenweiler, 2011). Now, in our first experiment, participants received feedback after retrieval practice in the form of the correct response. This might have been the factor underlying the short-term testing effect.

The feedback in Experiment 1 may also have prevented the interaction effect between study method and retention interval to occur. This is in line with the predictions of the bifurcation framework (Kornell et al., 2011). According to this framework, a test bifurcates the distribution of items’ memory strength: non-retrieved items remain low in strength while retrieved items become high in strength, resulting in a gap between the two sets of items. Furthermore, items that are retrieved during testing are strengthened more than items that are restudied. Because strong memories last, testing will result in better performance than restudying after a long interval. Feedback, however, also boosts the memory strength of non-retrieved items, thereby preventing bifurcation to occur. In that case, testing with feedback will strengthen all tested items, giving rise to a benefit of testing after both a short-term and a long-term interval.

## Experiment 2

To answer the final test questions of Experiment 1 correctly, participants in the retrieval practice condition had to apply the key information that had been provided as feedback. This may have granted an advantage to participants in the retrieval practice condition compared to the reread condition. Hence, our results, as well as those of Butler’s (2010) third experiment, might have been partly driven by this focused exposure to key information rather than by retrieval practice *per se*. To test this alternative account of our results, we conducted a second experiment. This experiment was similar to the first, but now with an extra reread-plus-statements condition, besides the retrieval practice condition and the reread condition. In the retrieval practice condition, participants answered four cued recall questions per text and received feedback after each question. Participants in the reread-plus-statements condition first reread a text for 2 min, and then read four isolated statements that contained the same information as the feedback in the retrieval practice condition (cf. Butler, 2010, Experiment 2 on near transfer). In the reread condition, participants reread a text for 2 min. So, in all three conditions, participants received the same key information that was necessary to answer the final test questions. However, in the reread condition, the key information was presented together with the additional information that was in the text. By contrast, in both the retrieval practice condition and the reread-plus-statements condition, participants received focused exposure to the key information. We therefore expected the difference between the retrieval practice condition and the reread-plus-statements condition on the final transfer test to be considerably smaller (or perhaps even absent) than the difference between retrieval practice and rereading.

Note that we decided to drop the 5-min condition in the second experiment, because the short-term final test results in the first experiment might have suffered from participants’ fatigue. A convenient side effect of this choice was that we had more participants – and hence more power – to detect an effect of our experimental manipulation on transfer after a retention interval of 1 week.

### Method

#### Participants

This experiment was carried out in accordance with the recommendations of the Ethical Committee of the Department DPECS at the Erasmus University Rotterdam, with written informed consent from all participants. Fifty-five people participated in this study and were rewarded with course credits. One participant was removed because she said she had only paid attention to the texts with the questions (retrieval practice condition), leaving a total number of 54 participants. Their mean age was 20.00 (*SD* = 4.10). Twenty of them were males, 34 were females. All of the participants were Psychology undergraduates (see Experiment 1). Twenty-two of them had the Dutch nationality, while the others had (15) other nationalities.

#### Materials and Design

Butler’s (2010) six prose texts and test questions were used again, see Experiment 1. The experiment had a 3 Study Method (reread vs. retrieval practice vs. reread-plus-statements) within-subjects design. We used a Latin Square to create nine counterbalance conditions, using three sets of two texts and six orders of initial study conditions. Following Simmons et al. (2011), we have reported all conditions and all measures in this experiment.

#### Procedure

The experiment was conducted on a computer using E-Prime software. As in Experiment 1 we used Butler’s (2010) original files, but now with some adjustments to include the extra reread-plus-statements condition. The procedure was identical to that in Experiment 1, except for the elimination of the 5-min retention interval and the new reread-plus-statements condition (and, as a result, two instead of three texts per study method). In the reread condition, two texts were reread three times. In the retrieval practice condition, participants took three identical four-item cued recall tests with immediate feedback on two other texts. This feedback was identical to that in Butler (2010) and in our Experiment 1. In the reread-plus-statements condition, participants repeatedly (i.e., three times) reread two of the texts, each followed by four statements that contained the same information as was presented as feedback in the retrieval practice condition, except that it was rephrased in order to make sense as prose (cf. the isolated sentences in Butler, 2010, Experiment 2). For example, one of the questions in the retrieval practice condition was the following: “A bat has a very different wing structure from a bird. What is the wing structure of a bat like relative to that of a bird?” The answer to this question was as follows: “A bird’s wing has fairly rigid bone structure that is efficient at providing lift, whereas a bat has a much more flexible wing structure that allows for greater maneuverability.” In the reread-plus-statements condition, the corresponding key statement was the following: “A bat has a very different wing structure from a bird. A bird’s wing has fairly rigid bone structure that is efficient at providing lift, whereas a bat has a much more flexible wing structure that allows for greater maneuverability.” Hence, the key statements contained part of the corresponding questions in order to be comprehensible. The instruction for the key statements was as follows: “Next, you will see four short pieces of information about the subject of the text that you have just read. Please read them carefully.” No time limit was given to read the key statements. One week later, participants returned to take the self-paced final test that consisted of 24 transfer questions on different knowledge domains.

### Results

#### Scoring

One research assistant and the first author independently scored 15% of the cued recall questions from the initial learning session. Each answer was scored as either correct or incorrect. Cohen’s kappa was used as the interrater reliability measure and was 0.67. This indicates a substantial level of agreement (Landis and Koch, 1977). The Pearson correlation coefficient between the total scores given by the two raters was *r* = 0.79, *p* = 0.021. All the remaining questions were scored by the first author.

Ten participants had coincidentally pushed Enter when they wanted to answer the first question of the final test, thereby going directly to the second question on the next screen. These 10 questions were treated as missing, and their values were estimated by taking the average of the scores on the other three questions corresponding to the same text.

#### Retrieval Practice Tests

The proportion of correct responses to the initial cued recall tests increased in a curvilinear fashion from Test 1 (*M* = 0.48, *SD* = 0.23) to Test 2 (*M* = 0.79, *SD* = 0.19) to Test 3 (*M* = 0.90, *SD* = 0.13). A repeated measures ANOVA yielded a statistical effect of Test, *F*(1.82,94.79) = 128.23, *MSE* = 0.02, *p* < 0.001, $\eta_{p}^{2}$ = 0.71, for which there was a linear trend, *F*(1,52) = 176.77, *MSE* = 0.03, *p* < 0.001, $\eta_{p}^{2}$ = 77, as well as a quadratic trend *F*(1,52) = 31.16, *MSE* = 0.01, *p* < 0.001, $\eta_{p}^{2}$ = 0.38. Note that due to an error, the retrieval practice data and time-on-task data of one participant were not included.

#### Time on Task

During retrieval practice, there were two texts with four questions each. The questions were repeated three times, resulting in a total number of 24 questions. The distributions of number of seconds spent on reading and answering questions were skewed to the right, so we report both the mean and the median. The average number of seconds that participants spent on answering a question during cued recall was 63.40 s (*SD* = 26.26), median 57.86 s. The mean number of seconds that participants spent on reading the feedback was 12.24 (*SD* = 5.61), median 10.90. Taken together, the mean number of seconds that participants spent on each question (responding and reading feedback) was 75.64 (median = 68.76). Because there were four questions per passage, it took participants 302.56 s on average to complete a test on each passage (median = 275.04 s). Furthermore, in the reread-plus-statements condition, there were two texts that were both followed by four statements. Participants read the texts and the statements three times, coming down to 24 statements in total. The mean number of seconds that participants spent on reading a statement was 20.44 (*SD* = 11.59), median 16.30, and they spent 120 s on rereading a passage. Because there were four statements per passage, this resulted in a total time per passage of 201.76 s (median 185.20 s). In the reread condition, all participants had 120 s to reread a passage.

#### Final Tests

A 3 Study Method (reread vs. retrieval practice vs. reread-plus-statements) Repeated Measures ANOVA on the proportion of correct answers on the final transfer test revealed a statistical main effect of study method, *F*(2,106) = 22.62, *MSE* = 0.04, *p* < 0.001, $\eta_{p}^{2}$ = 0.30 (**Table 2**). Bonferroni corrected pairwise comparisons revealed statistical differences between all three conditions. Retrieval practice differed from reread-plus-statements, *t*(53) = 3.08, *p* = 0.009, *r* = 0.39, 95% CI of the difference [0.02, 0.20]. In addition, there was a difference between retrieval practice and reread, *t*(53) = 6.26, *p* < 0.001, *r* = 0.65, 95% CI of the difference [0.15, 0.34]. Also, reread-plus-statements differed from reread, *t*(53) = 3.80, *p* = 0.001, *r* = 0.46, 95% CI of the difference [0.05, 0.22].

**Table 2:** Proportion of Answers Correct in Experiment 2 by Study Method.

Study method	
Retrieval practice	0.64 (0.03)
Reread-plus-statements	0.53 (0.03)
Reread	0.40 (0.03)


*Standard errors are between brackets.*

**FIGURE 1:**
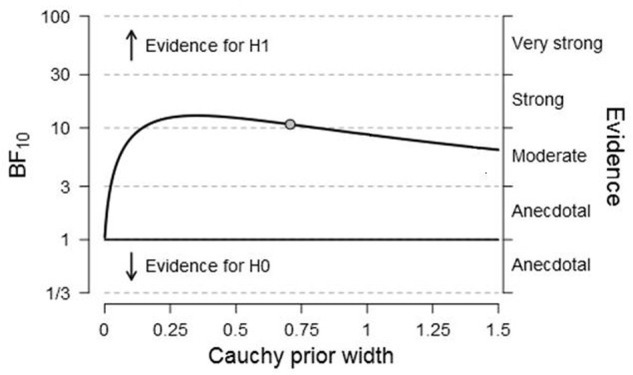
**Bayes Factor for the comparison between retrieval practice and reread-plus-statements (Experiment 2) as a function of the scale parameter *r* of the Cauchy prior for effect size under the alternative hypothesis.** The dot indicates the used prior width of *r* = 0.71. Figure adjusted from JASP, jasp-stats.org.

In addition, we performed a Bayesian Repeated Measures ANOVA (Rouder et al., 2012, 2016) in the software program JASP (Love et al., 2015; Wagenmakers et al., 2016) with a default uniform distribution of prior model probabilities. The Bayes Factor BF10 for the factor study method (reread vs. retrieval practice vs. reread-plus-statements) was larger than 100, indicating that the observed data were more than 100 times more likely under the alternative hypothesis than under the null hypothesis. According to Wetzels and Wagenmakers (2012), this is decisive evidence in favor of the alternative hypothesis that postulates a difference in means between the three conditions. As follow-up tests, we performed three new two-sided Bayesian paired samples *T*-Tests (Rouder et al., 2009) with a default Cauchy prior width of *r* = 0.71 for effect size on the alternative hypothesis. The comparison between retrieval practice and rereading delivered a Bayes factor of larger than 100, which is decisive evidence for the hypothesis that there is a difference between retrieval practice and rereading. In this experiment, there was a large benefit of retrieval practice compared to rereading. The comparison between reread-plus-statements and reread produced a Bayes factor of BF10 = 86.71, showing that the likelihood of the data under the alternative hypothesis was 86.71 times the likelihood of the data under the null hypothesis. This presents very strong evidence for the alternative hypothesis that there is a difference, in this case a large advantage of reread-plus-statements compared to reread. Finally, retrieval practice was compared to reread-plus-statements. This yielded a Bayes factor of BF10 = 10.77, meaning that the observed data were 10.77 times more likely under the alternative hypothesis than under the null hypothesis. This is strong evidence for the alternative hypothesis of a difference between conditions, in this case an advantage of retrieval practice over reread-plus-statements. To inspect the robustness of the latter analysis, the Bayes factor is plotted as a function of the scale parameter *r* of the Cauchy prior in **Figure 1**. As the scale parameter *r* increases (i.e., the prior becomes wider), the evidence for the alternative hypothesis gets weaker. However, even under the prior settings that least favor the alternative hypothesis, the Bayes factor is still larger than 6.50, indicating substantial evidence for the alternative hypothesis (Wetzels and Wagenmakers, 2012).

Again, the time-on-task differed considerably between the three conditions in Experiment 2. To assess whether this variable confounded the final test results, we calculated for each participant the time-on-task differences for each of the three unique condition combinations (with time-on-task in the reread condition being a constant). Furthermore, for each participant we calculated the final test difference scores for each of the three condition combinations. Subsequently we correlated these time-on-task difference scores with the relevant final test difference scores. For ‘retrieval practice/reread’, there was no statistical correlation between time-on-task differences and final test differences, *r* = 0.09, *p* = 0.525. The same applied to ‘reread-plus-statements/reread’, *r* = 0.01, *p* = 0.920, and to ‘retrieval practice/reread-plus-statements’, *r* = 0.08, *p* = 0.569. These non-statistical correlations indicate that the final test results were not confounded by time-on-task differences.

### Discussion

The results of Experiment 2 showed that the reread-plus-statements condition resulted in a higher score on the final transfer test than the reread condition. In addition, retrieval practice led to better performance on the final test than reread and better than reread-plus-statements. When retrieval practice was compared to reread-plus-statements, however, the effect size was much smaller than when it was compared to reread (resp., *r* = 0.39 versus *r* = 0.65); the proportion of explained variance fell by about 65% when retrieval practice was contrasted with reread-plus-statements (testing effect magnitude of *r*^2^ = 0.15) instead of reread (testing effect magnitude of *r*^2^ = 0.42). Taken together, the results of Experiment 2 suggest that the advantage of retrieval practice, found in Butler (2010) and in our first experiment, was partly due to the focused exposure to key information (i.e., the feedback). However, the advantage of retrieval practice over the reread-plus-statements condition indicates that practicing retrieval added something extra, above and beyond providing participants with focused exposure to key information.

## General Discussion

In Experiment 1, we replicated the third experiment of Butler’s study (Butler, 2010). Retrieval practice produced better performance than rereading on the final transfer test administered after 1 week, and also after 5 min. Experiment 2 was similar to Experiment 1, but with an extra reread-plus-statements condition. In this condition, the retrieval practice questions were replaced by rereading, followed by focused exposure to the key information. These key statements contained the same information as the feedback that participants received in the retrieval practice condition. In this manner, we examined whether the focused exposure to key information could explain the large advantage of retrieval practice over reread in Experiment 1.

In Experiment 2, retrieval practice again outperformed rereading on a delayed far transfer test. Moreover, transfer performance in the reread-plus-statements condition was considerably better than in the reread condition. In addition, retrieval practice resulted in a higher final test score than reread-plus-statements. However, the testing effect was much smaller when retrieval practice was compared to reread-plus-statements (*r* = 0.39, testing effect magnitude of *r*^2^ = 0.15, BF = 10.77) than when retrieval practice was compared to reread (*r* = 0.65, testing effect magnitude of *r^2^* = 0.42, BF > 100). Note that it is important to focus not only on the *p*-value but also on the size of the effect. Based on the *p*-values, one would simply conclude that retrieval practice leads to better far transfer than reread and reread-plus-statements. However, when the effect sizes are taken into account, a different picture emerges. That is, it becomes clear that the effect of retrieval practice might be partly attributed to the focused exposure to key information (i.e., the feedback). These findings are important from a theoretical as well as from a practical perspective. Both for theory development and for real-world applications (such as in educational practice), it is crucial to realize that the benefit of retrieval practice varies with the control condition to which it is compared.

Because time-on-task differed between conditions in both our experiments, we wanted to exclude the possibility that this variable was a confounder. We therefore inspected the correlations between time-on-task differences between conditions and final test differences. These correlations were all very small and statistically non-significant, indicating that there was no association between time-on-task and final test advantages of retrieval practice and reread-plus-statements over rereading. This seems in line with Butler’s (2010) study. In his second experiment, the time-on-task spent per text on the conceptual questions in the retrieval practice condition (168.4 s) was considerably *lower* than the time-on-task in the reread condition (240 s). Conversely, in his third experiment, the time-on-task in the reread condition was 120 s per text, and although the time-on-task in the retrieval practice condition was not reported, it is reasonable to assume that this was comparable to the time-on-task in the second experiment (168.4 s). In that case, the time-on-task in the retrieval practice condition in the third experiment was *higher* than the time-on-task in the reread condition. Still, in both experiments retrieval practice outperformed rereading on the final test. Together these findings indicate that time-on-task did not matter much for performance on the final transfer test that Butler and we used. This is in keeping with other studies where increased time-on-task was not related to retention performance (e.g., Amlund et al., 1986; Callender and McDaniel, 2009). Hence, we think that the time-on-task differences between conditions did not confound the final test results.

It might be argued that the benefit of retrieval practice in Experiment 2 was reduced compared to the reread-plus-statements condition because participants received less exposure to the key information in the retrieval practice condition than in the reread-plus-statements condition. In the reread-plus-statements condition, participants were exposed to the key information twice: first when rereading the text and second when reading the isolated statements. By contrast, one could assert that participants in the retrieval practice condition only were exposed to the key information twice when the material was successfully retrieved. However, some research (e.g., Kornell et al., 2009; Richland et al., 2009) suggests that retrieval attempts promote learning even when the attempts are unsuccessful. Moreover, in the vast majority of the testing effect studies, exposure to the to-be-learned information is lower after retrieval practice than after restudying because retrieval practice is not perfect. Nevertheless, large testing effects are observed in these studies on delayed final tests (e.g., Roediger and Karpicke, 2006b).

In addition, one could argue that the focused exposure to key information reduced the beneficial effect of retrieval practice compared to reread-plus-statements because the exposure to key information was more spaced (i.e., distributed over time) in the latter than in the former. This is because in the retrieval practice condition, the key information was provided as feedback immediately following the questions to which participants had to respond (massed repetition), whereas participants in the reread-plus-statements condition received the information after rereading the text (more spaced repetition). As a consequence, transfer performance in the reread-plus-statements condition might have benefitted from a spacing effect (e.g., Cepeda et al., 2006) and this – rather than focused exposure to key information *per se* – might have resulted in a smaller retrieval practice advantage.

Although the above line of reasoning is correct, it is only directed at repetitions *within* a relearning session, but does not take into account repetitions *across* the three relearning sessions. However, Karpicke and Bauernschmidt (2011) showed that when comparing repetition schedules on final test performance, it is pivotal to determine the absolute or total spacing per schedule. Consequently, in the present study, it is not appropriate to only focus on repetition *within* a relearning session (or only on repetitions *across* sessions, for that matter); instead, one should compare conditions on total spacing. This total spacing is obtained by combining spacing lags within and across learning sessions. In the present study, participants went through three relearning sessions in all conditions. Within a relearning session, repetition of key information was relatively massed in the retrieval practice condition and relatively spaced in the reread-plus-statements condition. However, the total spacing of repetitions (i.e., the combination of spacing within and across sessions) reveals a somewhat different picture. Consider a participant in the retrieval practice condition who correctly answers the first question in all three relearning sessions. This participant will be exposed to the key information six times (i.e., answer and feedback in each of the three sessions), coming down to five spacing intervals. Within a session, feedback is presented immediately after answering a question, resulting in a spacing interval of 0 s. However, given the time-on-task of one relearning session in the retrieval practice condition (i.e., answering the other three questions and reading the feedback), the next repetition in the second session appears after 226.92 s (3/4 ^∗^ 302.56). That is, the spacing from the first to the second session is 226.92 s. The same applies for the spacing between the second and the third session. Hence, in the retrieval practice condition, total spacing was 453.84 s. By contrast, in the reread-plus-statements condition, the total spacing between the six repetitions of the key information was about 392.64 s [2 ^∗^ (3/4 ^∗^ 201.76) + 3/4 ^∗^ 120].

So, in both the retrieval practice condition and the reread-plus-statements condition the exposure to the key information was spaced, but the conditions differ in total spacing. However, because the function between total spacing and memory performance reaches approximately an asymptote at a total spacing considerably shorter than those in our Experiment 2 (cf, Glenberg, 1976; Raaijmakers, 2003), it is unlikely that the difference in transfer test performance between the retrieval practice condition and the reread-plus-statements condition can be attributed to the total spacing difference. Still, the conditions differ on total spacing, and we cannot completely rule out the possibility that this variability –rather than the experimental manipulation– has confounded the final test results. Hence, future research might include a key statements condition that is modeled after the retrieval practice condition, i.e., with two massed exposures of key information without rereading the total text. Furthermore, the time-on-task should be held fixed and equated between conditions. In this way, total spacing will be equal between conditions, and possible final test differences can be exclusively attributed to the experimental manipulation.

A remaining question is why retrieval practice led to better performance than reread-plus-statements. This finding could be due to both indirect and direct effects that retrieval practice has on learning (Roediger and Karpicke, 2006b). An indirect effect means that the influence of retrieval practice is mediated by another factor, such as motivation. In our study, it is possible that during the retrieval attempt, participants became aware of what they did not yet know, causing them to pay more attention to the subsequent feedback. This process might have enhanced their final test scores. That would also explain why there was a short-term testing effect in our first study; in other studies where feedback was provided, a short-term benefit of retrieval practice occurred as well (e.g., Carrier and Pashler, 1992; Bishara and Jacoby, 2008; Carpenter et al., 2008; Jacoby et al., 2010; Kang, 2010; Wartenweiler, 2011). In addition to this indirect effect, retrieval practice might have exerted its influence in a direct way. By retrieving knowledge from memory, the knowledge itself is altered, thereby accommodating retrieval at a later point in time (Karpicke and Grimaldi, 2012).

Taken together, the current study shows that the testing effect in far transfer across different knowledge domains (Butler, 2010) is robust. We replicated the results of Butler’s (2010) third experiment with a comparable effect size, indicating that retrieval practice can greatly enhance performance on a far transfer test. However, our results also show that the success of retrieval practice was partly a matter of providing focused exposure to key information. When retrieval practice was compared to a condition that involved rereading the texts and then reading the key information in the form of isolated statements, the benefit of retrieval practice decreased to a fair extent. Hence, the focused exposure to key information (i.e., the feedback) seems be of crucial importance in the retrieval practice condition. Upcoming research could investigate the precise role of focused exposure to key information in the far transfer testing effect.

## Data Access

All data of this study can be retrieved from the Open Science Framework^2^.

## Ethics Statement

Ethical Committee DPECS, Erasmus University Rotterdam Participants granted their written informed consent before taking part in the study.

## Author Contributions

GvE, PV, and RR designed the experiment, MP and GvE collected and analyzed the data, GvE wrote, and PV, MP, and RR provided critical revisions on the manuscript, and GvE, PV, MP, and RR approved the version to be published.

## Conflict of Interest Statement

The authors declare that the research was conducted in the absence of any commercial or financial relationships that could be construed as a potential conflict of interest.

## Footnotes

1 Due to a miscalculation of the standard deviation, the reported *Cohen’s d* in Butler’s (2010) article was 0.99, but in reality it was 1.17 (personal correspondence, July 14, 2016).

2 https://osf.io/2h34r/

## References

[B1] Agarwal P. K. Karpicke J. D. Kang S. H. K. Roediger H. L. McDermott K. B. (2008). Examining the testing effect with open- and closed-book tests. *Appl. Cogn. Psychol.* 22 861–876. 10.1002/acp.1391

[B2] Amlund J. T. Kardash C. A. M. Kulhavy R. W. (1986). Repetitive reading and recall of expository text. *Read. Res. Q.* 21 49–58. 10.2307/747959

[B3] Barnett S. M. Ceci S. J. (2002). When and where do we apply what we learn? A taxonomy for far transfer. *Psychol. Bull.* 4 612–637. 10.1037//0033-2909.128.4.612 12081085

[B4] Bishara A. J. Jacoby L. L. (2008). Aging, spaced retrieval, and inflexible memory performance. *Psychon. Bull. Rev.* 15 52–57. 10.3758/PBR.15.1.52 18605479

[B5] Blunt J. R. Karpicke J. D. (2014). Learning with retrieval-based concept mapping. *J. Educ. Psychol.* 3 849–858. 10.1037/a0035934

[B6] Butler A. C. (2010). Repeated testing produces superior transfer of learning relative to repeated studying. *J. Exp. Psychol.* 5 1118–1133. 10.1037/a0019902 20804289

[B7] Callender A. A. McDaniel M. (2009). The limited benefits of rereading educational texts. *Contemp. Educ. Psychol.* 34 30–41. 10.1016/j.cedpsych.2008.07.001

[B8] Carpenter S. K. (2011). Semantic information activated during retrieval contributes to later retention: support for the mediator effectiveness hypothesis of the testing effect. *J.Exp. Psychol.* 37 1547–1552. 10.1037/a0024140 21707217

[B9] Carpenter S. K. (2012). Testing enhances the transfer of learning. *Curr. Dir. Psychol. Sci.* 21 279–283. 10.1177/0963721412452728

[B10] Carpenter S. K. DeLosh E. (2006). Impoverished cue support enhances subsequent retention: support for the elaborative retrieval explanation of the testing effect. *Mem. Cogn.* 34 268–276. 10.3758/BF03193405 16752591

[B11] Carpenter S. K. Pashler H. Cepeda N. J. (2009). Using tests to enhance 8th grade students’ retention of U. S. history facts. *Appl. Cogn. Psychol.* 23 760–771. 10.1002/acp.1507

[B12] Carpenter S. K. Pashler H. Vul E. (2006). What types of learning are enhanced by a cued recall test? *Psychon. Bull. Rev.* 13 826–830. 10.3758/BF03194004 17328380

[B13] Carpenter S. K. Pashler H. Wixted J. T. Vul E. (2008). The effect of tests on learning and forgetting. *Mem. Cogn.* 36 438–448. 10.3758/MC.36.2.438 18426072

[B14] Carrier M. Pashler H. (1992). The influence of retrieval on retention. *Mem. Cogn.* 20 632–642. 10.3758/BF03202713 1435266

[B15] Cartwright C. (1991). Replicability, reproducibility, and robustness: comments on Harry Collins. *Hist. Polit. Econ.* 23 143–155. 10.1215/00182702-23-1-143

[B16] Cepeda N. J. Pashler H. Vul E. Wixted J. T. Rohrer D. (2006). Distributed practice in verbal recall tasks: a review and quantitative synthesis. *Psychol. Bull.* 132 354–380. 10.1037/0033-2909.132.3.354 16719566

[B17] Chan J. C. K. McDermott K. B. Roediger H. L. (2006). Retrieval-induced facilitation: initially nontested material can benefit from prior testing of related material. *J. Exp. Psychol.* 4 553–571. 10.1037/0096-3445.135.4.553 17087573

[B18] Coppens L. C. Verkoeijen P. P. J. L. Rikers R. M. J. P. (2011). Learning Adinkra symbols: the effect of testing. *J. Cogn. Psychol.* 23 351–357. 10.1080/20445911.2011.507188

[B19] Cumming G. (2014). The new statistics: why and how. *Psychol. Sci.* 25 7–29. 10.1177/0956797613504966 24220629

[B20] Delaney P. F. Verkoeijen P. P. J. L. Spirgel A. S. (2010). “Spacing and testing effects: a deeply critical, lengthy, and at times discursive review of the literature,” in *The Psychology of Learning & Motivation* Vol. 53 ed. Ross B. H. (Burlington, VT: Academic Press), 63–147.

[B21] Dienes Z. (2011). Bayesian versus orthodox statistics: which side are you on? *Perspect. Psychol. Sci.* 6 274–290. 10.1177/1745691611406920 26168518

[B22] Dunlosky J. Rawson K. A. Marsh E. J. Nathan M. J. Willingham D. T. (2013). Improving students’ learning with effective learning techniques: promising directions from cognitive and educational psychology. *Psychol. Sci. Public Interest* 14 4–58. 10.1177/1529100612453266 26173288

[B23] Glenberg A. M. (1976). Monotonic and nonmonotonic lag effects in paired-associate and recognition memory paradigms. *J. Verbal Learn. Verbal Behav.* 15 1–16. 10.1016/S0022-5371(76)90002-5

[B24] Halamish V. Bjork R. A. (2011). When does testing enhance retention? A distribution- based interpretation of retrieval as a memory modifier. *J. Exp. Psychol.* 37 801–812. 10.1037/a0023219 21480751

[B25] Hinze S. R. Wiley J. (2011). Testing the limits of testing effects using completion tests. *Memory* 19 290–304. 10.1080/09658211.2011.560121 21500089

[B26] Ioannidis J. P. A. (2005). Why most published research findings are false. *PLoS Med.* 2:e124 10.1371/journal.pmed.0020124 PMC1182327 16060722

[B27] Jacoby L. L. Wahlheim C. N. Coane J. H. (2010). Test-enhanced learning of natural concepts: effects on recognition memory, classification, and metacognition. *J. Exp. Psychol.* 36 1441–1451. 10.1037/a0020636 20804279

[B28] Johnson C. I. Mayer R. E. (2009). A testing effect with multimedia learning. *J. Educ. Psychol.* 3 621–629. 10.1037/a0015183

[B29] Kang S. H. K. (2010). Enhancing visuospatial learning: the benefit of retrieval practice. *Mem. Cogn.* 38 1009–1017. 10.3758/MC.38.8.1009 21156865

[B30] Kang S. H. K. McDermott K. B. Roediger H. L. (2007). Test format and corrective feedback modify the effect of testing of long-term retention. *Eur. J. Cogn. Psychol.* 19 528–558. 10.1080/09541440601056620

[B31] Karpicke J. D. (2012). Retrieval-based learning: active retrieval promotes meaningful learning. *Curr. Dir. Psychol. Sci.* 21 157–163. 10.1177/0963721412443552

[B32] Karpicke J. D. Bauernschmidt A. (2011). Spaced retrieval: absolute spacing enhances learning regardless of relative spacing. *J. Exp. Psychol.* 37 1250–1257. 10.1037/a0023436 21574747

[B33] Karpicke J. D. Grimaldi P. J. (2012). Retrieval-based learning: a perspective for enhancing meaningful learning. *Educ. Psychol. Rev.* 24 401–418. 10.1007/s10648-012-9202-2

[B34] Karpicke J. D. Lehman M. Aue W. R. (2014). “Retrieval-based learning: an episodic context account,” in *The Psychology of Learning & Motivation* Vol. 61 ed. Ross B. H. (San Diego, CA: Elsevier Academic Press), 237–284. 10.1016/B978-0-12-800283-4.00007-1

[B35] Karpicke J. D. Zaromb F. M. (2010). Retrieval mode distinguishes the testing effect from the generation effect. *J. Mem. Lang.* 62 227–239. 10.1016/j.jml.2009.11.010

[B36] Klein R. A. Ratliff K. A. Vianello M. Adams R. B. Jr. Bahník Š Bernstein M. J. (2014). Investigating variation in replicability: a “many labs” replication project. *Soc. Psychol.* 45 142–152. 10.1027/1864-9335/a000178

[B37] Kline R. B. (2004). *Beyond Significance Testing. Reforming Data Analysis Methods in Behavioral Research.* Washington, DC: APA Books.

[B38] Kornell N. Bjork R. A. Garcia M. A. (2011). Why tests appear to prevent forgetting: a distribution-based bifurcation model. *J. Mem. Lang.* 65 85–97. 10.1016/j.jml.2011.04.002

[B39] Kornell N. Hays M. J. Bjork R. A. (2009). Unsuccessful retrieval attempts enhance subsequent learning. *J. Exp. Psychol. Learn. Mem. Cogn.* 35 989–998. 10.1037/a0015729 19586265

[B40] Landis J. R. Koch G. G. (1977). The measurement of observer agreement for categorical data. *Biometrics* 33 159–174. 10.2307/2529310 843571

[B41] Love J. Selker R. Marsman M. Jamil T. Dropmann D. Verhagen A. J. (2015). *JASP (Version 0.7.1.12) [Computer software].* Amsterdam: JASP Project.

[B42] Lyle K. B. Crawford N. A. (2011). Retrieving essential material at the end of lectures improves performance on statistics exams. *Teach. Psychol.* 38 94–97. 10.1177/0098628311401587

[B43] McDaniel M. A. Howard D. C. Einstein G. O. (2009). The read-recite-review study strategy: effective and portable. *Psychol. Sci.* 20 516–522. 10.1111/j.1467-9280.2009.02325.x 19320858

[B44] McDaniel M. A. Thomas R. C. Agarwal P. K. McDermott K. B. Roediger H. L. (2013). Quizzing in middle-school science: successful transfer performance on classroom exams. *Appl. Cogn. Psychol..* 27 360–372. 10.1002/acp.2914

[B45] McDermott K. B. Agarwal P. K. D’Antonio L. Roediger H. L. McDaniel M. A. (2014). Both multiple-choice and short-answer quizzes enhance later exam performance in middle and high school classes. *J. Exp. Psychol.* 20 3–21. 10.1037/xap0000004 24274234

[B46] Open Science Collaboration (2012). An open, large-scale, collaborative effort to estimate the reproducibility of psychological science. *Perspect. Psychol. Sci.* 7 657–660. 10.1177/1745691612462588 26168127

[B47] Pashler H. Wagenmakers E.-J. (2012). Editors’ introduction to the special section on replicability in psychological science: a crisis of confidence? *Perspect. Psychol. Sci.* 7 528–530. 10.1177/1745691612465253 26168108

[B48] Raaijmakers J. G. W. (2003). Spacing and repetition effects in human memory: application of the SAM model. *Cogn. Sci.* 27 431–452. 10.1016/S0364-0213(03)00007-7

[B49] Rawson K. A. Vaughn K. E. Carpenter S. K. (2015). Does the benefit of testing depend on lag, and if so, why? Evaluating the elaborative retrieval hypothesis. *Mem. Cogn.* 43 619–633. 10.3758/s13421-014-0477-z 25344296

[B50] Richland L. E. Kornell N. Kao L. S. (2009). The pretesting effect: do unsuccessful retrieval attempts enhance learning? *J. Exp. Psychol.* 15 243–257. 10.1037/a0016496 19751074

[B51] Roediger H. L. Agarwal P. K. McDaniel M. A. McDermott K. M. (2011). Test-enhanced learning in the classroom: long-term improvements from quizzing. *J. Exp. Psychol.* 17 382–395. 10.1037/a0026252 22082095

[B52] Roediger H. L. Butler A. C. (2011). The critical role of retrieval practice in long-term retention. *Trends Cogn. Sci.* 15 20–27. 10.1016/j.tics.2010.09.003 20951630

[B53] Roediger H. L. Karpicke J. D. (2006a). Test-enhanced learning: taking memory tests improves long-term retention. *Psychol. Sci.* 17 249–255. 10.1111/j.1467-9280.2006.01693.x 16507066

[B54] Roediger H. L. Karpicke J. D. (2006b). The power of testing memory: basic research and implications of educational practice. *Perspect. Psychol. Sci.* 1 181–208. 10.1111/j.1745-6916.2006.00012.x 26151629

[B55] Rohrer D. Taylor K. Sholar B. (2010). Tests enhance the transfer of learning. *J. Exp. Psychol.* 36 233–239. 10.1037/a0017678 20053059

[B56] Rouder J. N. Morey R. D. Speckman P. L. Province J. M. (2012). Default Bayes factors for ANOVA designs. *J. Math. Psychol.* 56 356–374. 10.1016/j.jmp.2012.08.001

[B57] Rouder J. N. Morey R. D. Verhagen A. J. Swagman A. R. Wagenmakers E.-J. (2016). Bayesian analysis of factorial designs. *Psychol. Methods* 10.1037/met0000057 [Epub ahead of print]. 27280448

[B58] Rouder J. N. Speckman P. L. Sun D. Morey R. D. Iverson G. (2009). Bayesian t tests for accepting and rejecting the null hypothesis. *Psychon. Bull. Rev.* 16 225–237. 10.3758/PBR.16.2.225 19293088

[B59] Rowland C. A. (2014). The effect of testing versus restudy on retention: a meta-analytic review of the testing effect. *Psychol. Bull.* 140 1432–1463. 10.1037/a0037559 25150680

[B60] Rowland C. A. DeLosh E. L. (2015). Mnemonic benefits of retrieval practice at short retention intervals. *Memory* 23 403–419. 10.1080/09658211.2014.889710 24579674

[B61] Salomon G. Perkins D. N. (1989). Rocky roads to transfer: rethinking mechanisms of a neglected phenomenon. *Educ. Psychol.* 24 113–142. 10.1207/s15326985ep2402_1

[B62] Schmidt S. (2009). Shall we really do it again? The powerful concept of replication is neglected in the social sciences. *Rev. Gen. Psychol.* 13 90–100. 10.1037/a0015108

[B63] Sensenig A. E. Littrell-Baez M. K. DeLosh E. L. (2011). Testing effects for common versus proper names. *Memory* 19 664–673. 10.1080/09658211.2011.599935 21919593

[B64] Simmons J. P. Nelson L. D. Simonsohn U. (2011). False-positive psychology: undisclosed flexibility in data collection and analysis allows presenting anything as significant. *Psychol. Sci.* 22 1359–1366. 10.1177/0956797611417632 22006061

[B65] Wagenmakers E.-J. Love J. Marsman M. Jamil T. Ly A. Verhagen A. J. (2016). *Bayesian Inference for Psychology. Part II: Example Applications with JASP.* Available at: https://osf.io/ahhdr/ [accessed November 2, 2016]. 10.3758/s13423-017-1323-7 PMC5862926 28685272

[B66] Wartenweiler D. (2011). Testing effect for visual-symbolic material: Enhancing the learning of Filipino children of low socio-economic status in the public school system. *Int. J. Res. Rev.* 6 74–93.

[B67] Wetzels R. Wagenmakers E.-J. (2012). A default Bayesian hypothesis test for correlations and partial correlations. *Psychon. Bull. Rev.* 19 1057–1064. 10.3758/s13423-012-0295-x 22798023 PMC3505519

[B68] Wooldridge C. L. Bugg J. M. McDaniel M. A. Liu Y. (2014). The testing effect with authentic educational materials: a cautionary note. *J. Appl. Res. Mem. Cogn.* 3 214–221. 10.1016/j.jarmac.2014.07.001

